# Food taboos and animal conservation: a systematic review on how cultural expressions influence interaction with wildlife species

**DOI:** 10.1186/s13002-023-00600-9

**Published:** 2023-07-15

**Authors:** André Santos Landim, Jeferson de Menezes Souza, Lucrécia Braz dos Santos, Ernani Machado de Freitas Lins-Neto, Daniel Tenório da Silva, Felipe Silva Ferreira

**Affiliations:** 1 Programa de Pós-Graduação em Ciências da Saúde e Biológicas, Universidade Federal do Vale do São Francisco (UNIVASF), Petrolina, Pernambuco Brazil; 2 Programa de Pós-Graduação em Biotecnologia, Universidade Estadual de Feira de Santana (UEFS), Feira de Santana, Bahia Brazil; 3 Núcleo de Estudos de Conservação da Caatinga (NECC)/Colegiado de Ecologia, Universidade Federal do Vale do São Francisco (UNIVASF), Petrolina, Pernambuco Brazil; 4 Programa de Pós-Graduação em Ecologia Humana e Gestão Socioambiental, Universidade Do Estado da Bahia (UNEB), Juazeiro, Bahia Brazil; 5 Programa de Pós-Graduação em Ecologia e Evolução, Universidade Estadual de Feira de Santana (UEFS), Feira de Santana, Bahia Brazil

**Keywords:** Conservation, Dietary restrictions, Sociocultural systems, Wild animals

## Abstract

**Background:**

Human societies have food taboos as social rules that restrict access to a particular animal. Taboos are pointed out as tools for the conservation of animals, considering that the presence of this social rule prevents the consumption of animals. This work consists of a systematic review that aimed to verify how food taboos vary between different animal species, and how this relationship has influenced their conservation.

**Methods:**

For this systematic review, the search for articles by keywords took place in the databases “Science Direct,” Scopus,” “SciELo” and “Web of Science,” associating the term “taboo” with the taxa “amphibians,” “birds,” “mammals,” “fish” and “reptiles.” From this search, 3959 titles were found related to the key terms of the research. After the entire screening process carried out by paired reviewers, only 25 articles were included in the search.

**Results:**

It was identified that 100 species of animals are related to some type of taboo, and segmental taboos and specific taboos were predominant, with 93 and 31 citations, respectively. In addition, the taxon with the most taboos recorded was fish, followed by mammals. Our findings indicate that the taboo protects 99% of the animal species mentioned, being a crucial tool for the conservation of these species.

**Conclusions:**

The present study covered the status of current knowledge about food taboos associated with wildlife in the world. It is noticeable that taboos have a considerable effect on animal conservation, as the social restrictions imposed by taboos effectively contribute to the local conservation of species.

**Supplementary Information:**

The online version contains supplementary material available at 10.1186/s13002-023-00600-9.

## Background

The process of eating is influenced by social, cultural and biological factors, leading human populations to select certain foods and avoid others. People recognize and classify foods for their nutrition, considering preferences that determine the intensity and frequency with which certain resources are consumed [1–3].

About dietary restrictions, taboos stand out as an important cultural element in several societies [4–7]. Food taboos are cultural elements that represent unwritten rules regulating human behavior toward certain resources, appearing in two forms: general taboos, which are imposed on an entire ethnic group making them never eat certain foods, and specific taboos, which are understood as temporary and interfere with a period of the individual's life, such as dietary restrictions at certain ages, in the face of illnesses and at certain times of life [8, 9].

Food taboos act by preventing access to a particular food resource, and several characteristics are related to define a species as taboo. Animals may be avoided as food due to the presence of toxicity, parasites, fat content, position in the food chain they occupy, microhabitat and their conservation status [10]. In a case study in Brazil, it was found that the existing dietary restrictions among fishermen populations in the southeast region were related to the shape of the fish, its appearance, odor, behavior, conspicuous teeth, absence of scales, strong or heavy meat (called in Brazil “reimosa”), habit of eating slime and presence of blood [11].

Additionally, aspects related to the local availability of fauna (considering the richness and abundance of species) and access to other proteins are pointed out as motivators for the absence or presence of food taboos. The literature shows cases in which the food resource decreases, there is a tendency to make food taboos more flexible [4, 9].

The presence of a food taboo in a human society brings a debate associated with fauna conservation. The defended hypothesis is that dietary restrictions result in adaptive strategies that contribute to the conservation and management of natural resources, above all, protecting some species of animals [12]. In this sense, the literature suggests that the presence of taboos directly contributes to the conservation of animal species [4, 13, 14]. However, there is a lack of studies that show whether in fact food taboos act as cultural elements that contribute to the conservation of fauna. Furthermore, there are gaps in knowledge about how food taboos behave in relation to taxonomic groups (birds, mammals, reptiles and amphibians), and how they appear in different regions of the planet.

Thus, the present study aimed to carry out a systematic review based on the following motivating questions: (1) Do food taboos influence fauna conservation? and (2) is there variation in the types of taboos between taxonomic groups and continents?

## Material and methods

### Research strategy and selection of studies

The systematic review was performed based on the Cochrane Handbook for Systematic Reviews of Interventions guideline and the Preferred Reporting Items for Systematic Reviews and Meta-Analyses (PRISMA) tool. Potentially relevant studies were identified through a search of Scopus, SciELO, Web of Science and Science Direct databases. The following research questions were used for this research: Do food taboos influence the conservation of wild species? and Do taboos influence fauna protection attitudes vary between taxonomic groups and continents?

As a search strategy, the standardized term “Taboo” was used, combined with terms related to animal taxa “Mammals,” “Reptiles,” “Amphibians,” “Birds” and “Fish,” linked by the Boolean operator “*and*.” These terms are considered standardized because they were selected from consultations in the encyclopedia of controlled vocabularies in the “National Library of Medicines” through the “Medical Subject Headings” (MeSH) and in the VHL through the “Descriptors in Health Sciences” (DeCS). The search was performed using terms in English, Portuguese and Spanish. No time limit was used in the database search.

### Inclusion and exclusion criteria for studies

Studies that met the following eligibility criteria were included in the review: (1) publication in English, Spanish or Portuguese; (2) object of study refers to animals with associated food taboo and (3) study points out whether the taboo associated with the animal leads to death or not of the species. Works were excluded: (1) unavailable in full; (2) abstracts published in conference proceedings; (3) letter to the editor; (4) literature review; (5) integrative review; (6) scoping review; (7) systematic review with or without meta-analysis; (8) systematic review overview with or without meta-analysis; (9) book chapter; (10) dissertations; (11) theses; (12) studies with imprecise results in reaction to taboos associated with species and (13) articles without the scientific name of the animal.

According to the eligibility criteria, the articles were selected according to the evaluation of the titles, followed by readings of the abstracts. If the article was appropriate, it was read in full. The selection was carried out by two researchers (paired review), called Reviewer 1 and Reviewer 2. In situations of disagreement between the reviewers, a third reviewer performed the tiebreaker.

The initial screening of articles found in the databases was performed using the EndNote software.^x9^ to exclude duplicate titles. Both the paired selection of titles and abstracts were performed using the Rayyan a software [15]. To verify the degree of agreement between the reviewers, the Kappa test was applied. The Kappa coefficient can be defined as a measure of association used to test the degree of agreement (reliability and precision) between evaluators [16]. The interpretation of the magnitude of the concordance estimators is agreed as: 0 (absent), 0–0.19 (poor/insignificant), 0.21–0.39 (fair), 0.40–0.59 (moderate), 0.60–0.79 (substantial) and ≥ 0.80 (almost perfect) [17]. Kappa test calculations were performed using the IBM SPSS Statistics 20 software.

The tabulation of the data was performed in Microsoft® Excel®, registering the information of the articles such as author; year of publication; country; study design; duration of study; species name; gender; family; order and class and endemisms, and if food taboo leads to death or not.

### Data analysis

Data were analyzed qualitatively, taking into account the quality of the study, number of cited species, classification of taboos and classification of the species in relation to the threat of extinction according to the International Union for Conservation of Nature's (IUCN). The evaluation of the quality of the study was carried out through the analysis of the risk of bias in relation to: (1) sample size of the study, (2) indication of the area and population of the study, (3) species identification strategy, (4) data analysis and (5) exposure of food taboos (Table 1). Methodological quality assessment and risk of bias were performed using Review Manager (RevMan) 5.4. [18].

**Table 1:** Criteria used for bias analysis

Criteria for risk of bias analysis	Low risk of bias	Moderate risk of bias	High risk of bias
Study sample size*	Sample equal to the universe Sample randomness of 5% All heads of household interviewed Stabilized accumulation curve	Sample extracted from the universe with error above 5% and < 10% Representation of up to 80% of heads of household No population information, but stable accumulation curve	Sample extracted with > 10% error Representativeness < 80% of heads of household There is no information on the number of households Accumulation curve moves away from stabilization
Indication of the study area and population	Presents a map of the study area and detailed information on the study population	Not applicable	It does not present a map of the study area and detailed information on the study population
Species identification strategies	Use of photographs, specimen collection and field observation	Not applicable	Comparison of vernacular/local name with available literature
Data analysis	Creation of statistical models and quantitative indices	Qualitative analyses and descriptive statistics	Qualitative analyses
Exposition of food taboos	Clear definition of taboo motivations	Not applicable	Taboo tangential exposure

(*) Sample size analyzed according to the methodology proposed by Medeiros et al. [48], the table presents some criteria as examples, other criteria can be consulted in the original study cited in the references of this article

The number of animals cited was recorded by simple counting, considering the number of times an animal is mentioned in different works. The number of species consists of the frequency in which a species appears, without considering repetitions. For example, if a species is cited by two works in different countries or not, we compute that the “Number of animals” is equal to two and the “Number of species” is one. For the classification of food taboos, the classification by Colding and Folke [19, 47] was adopted, classifying them into “specific taboos,” “segmental taboos,” “method taboos,” “life history taboos,” “habitat taboos” and “time taboos.” It was also recorded whether the type of taboo was related to the death of the animal.

## Results

The search for articles in the databases returned a total of 46,117 titles related to the descriptors. A total of 12,705 articles were excluded for being duplicated, with 33,412 being included for title analysis. After reading the titles, a total of 29,453 articles were excluded because they did not meet the eligibility criteria. Of the 3959 remaining titles, 1362 studies were excluded, 448 because they dealt with taboos related to insects and 914 because they were not the object of study of this research.

**Fig. 1:**
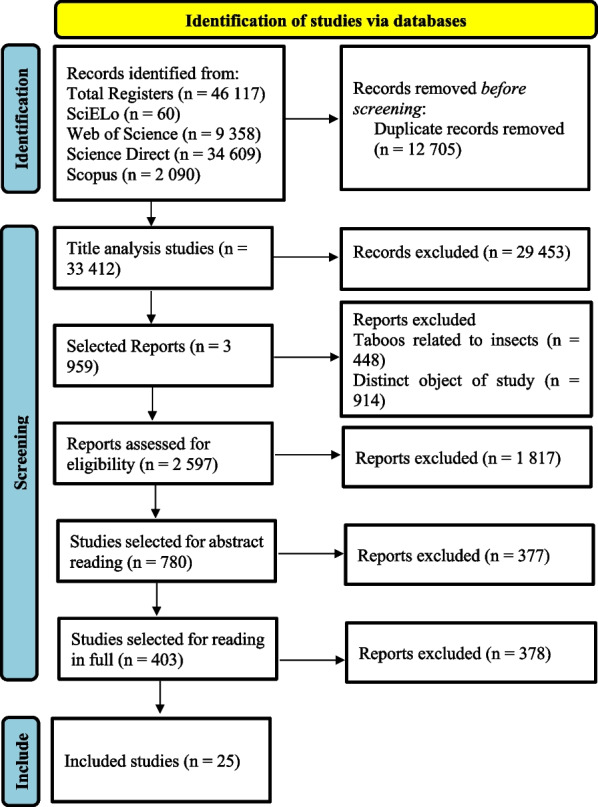
Studies identified by searching the databases, based on Page et al. [49]

Before selection by reading the abstracts, a third reviewer was asked to analyze the 2597 titles that passed the initial screening, 1817 articles being excluded. A total of 780 articles were included for reading the abstracts, 377 studies being excluded at this stage. A total of 403 articles were read in full, and 25 studies were included in this review (Fig. 1, see Additional file 1).

**Fig. 2:**
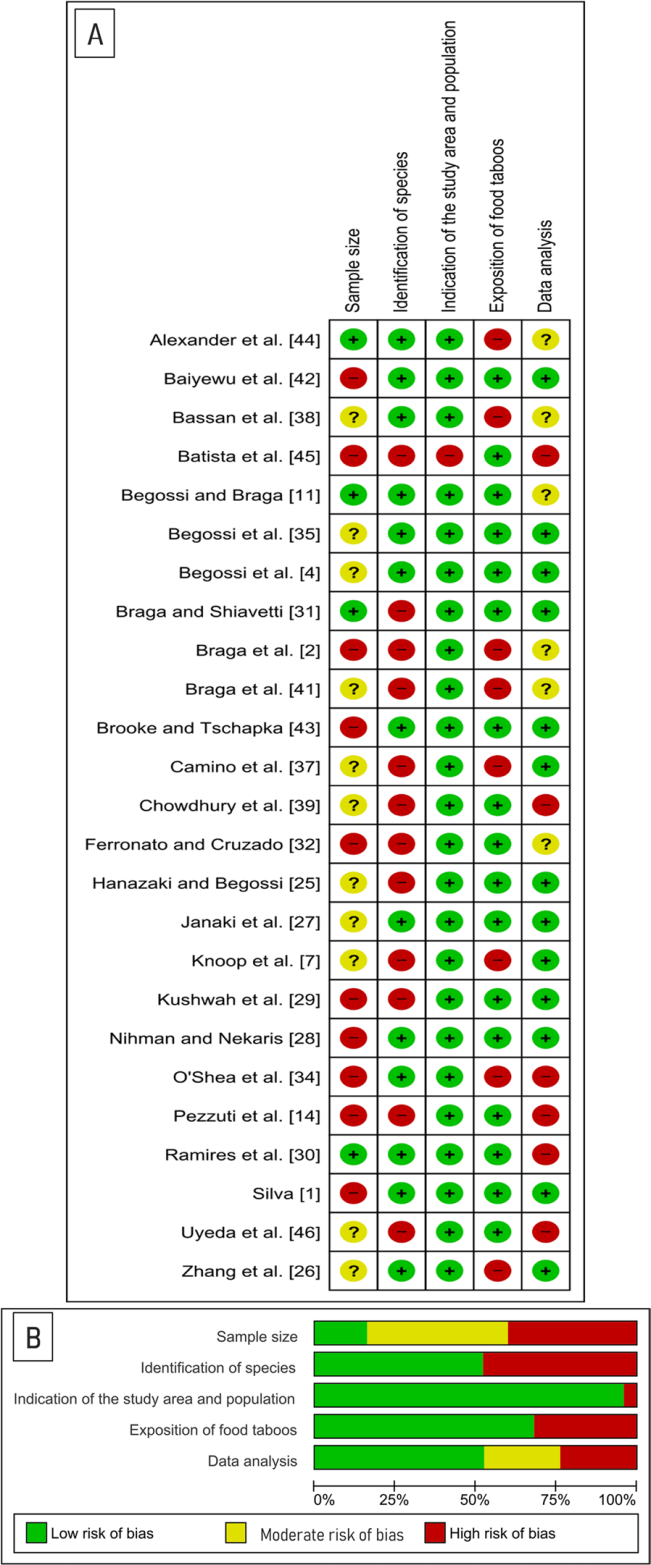
Authors' assessment of each risk of bias item for each scientific article included

The Kappa test indicated a reasonable agreement in the analysis of the titles (*k* = 0.309) and moderate agreement (*k* = 0.438) in the selection by reading the abstracts. Regarding the risk of bias, it was identified that 16% of the studies showed low risk of bias, 44% moderate risk and 40% high risk of bias in relation to the sample size. Regarding the identification of species, 52% of the works used photographs of the animals, collected parts or whole animals, presenting a low risk of bias. A total of 96% presented a good characterization of the study area and population, with maps of the area, geographic coordinates and cultural context. For the discussion of taboos, 64% showed low risk of bias, and 24% of the studies showed high risk of bias or moderate risk of bias for data analysis (Fig. 2).

A total of 130 animals distributed in 100 species were identified with some associated taboo. The species *Pseudoplatystoma fasciatum*, *Hoplias malabaricus* and *Chelonoidis denticulatus* presented the highest citation frequency, with four citations. It was registered that the taboo protects 99% of the registered species, avoiding the death of the animal. The only exception was the *Pteropus tonganus* present in Niue (Oceania), where a habitat taboo is associated with the death of the species. Regarding the taxonomic groups, fish had the greatest diversity of taboo species (44 species, average of five animals cited per study), followed by mammals (*n* = 35); reptiles (*n* = 16) and birds (five species) (Table 2).

**Table 2:** Identified animals and associated food taboos

Class/species	Country/continent	Global status	Taboo	Taboo description
*Mammals*				
*Nycticebus javanicus* (É. Geoffroy Saint-Hilaire, 1812)	Indonesia/Asia	EN	TE	Taking the animal home can cause misfortune, tragedies, natural catastrophes [28]
*Funambulus pennanti* (Wroughton, 1905)	India/Asia	LC	TE	The belief in a community that whoever kills the animal goes to hell [29]
*Pardofelis marmorata* (Martin, 1837)		NT	TE	They are not killed because they are totemic symbols for various tribes [46]
*Budorcas taxicolor* (Hodgson, 1850)		VU	TS; LST and TE	Consumption is restricted to the higher status population, there is a belief that the animal is descended from the Tibetan royal lineage [27]
*Panthera tigris* t*igris* (Linnaeus, 1758)		EN	TE	In the Mishmi tribe, it is believed that the species is ancestral sibling. Anyone who hunts this animal is penalized [27]
*Catopuma temminckii* (Vigors and Horsfiels, 1827)		NT	TE	They are not killed because they are totemic symbols for various tribes [46]
*Grus antigone* (Linnaeus, 1758)		VU	TE	Whoever kills the animal will be punished with the birth of handicapped children [29]
*Felis catus* (Linnaeus, 1758)		NE	TE	In the Bhil community, cat killing is prohibited, as whoever does so can go to hell [29]
*Trachypithecus pileatus* (Blyth, 1843)		VU	TE	Brings luck [27]
*Prionailurus viverrinus* (Bennett, 1833)	Bangladesh/Asia	VU	TE	Sacred animal [39]
*Hoolock tianxing* (Peng-Fei Fan, Kai He, Xing Chen et al., 2017)	China/Asia	EN	TE	They are considered ancestors or gods [26]
*Lagostomus maximus* (Desmarest, 1817)	Argentina/South America	LC	TS	Meat taste bad [37]
*Myrmecophaga tridactyla* (Linnaeus, 1758)		VU	TS	Pregnant women cannot eat [37]
*Mazama americana* (Erxleben, 1777)	Brazil/South America	DD	TE	Meat causes swelling of the eyes and stomach and causes dizziness [1]
*Ateles chamek* (Humboldt, 1812)		EN	TS	Aggravates inflammation [7]
*Cebus albifrons* (Humboldt, 1812)		EN	TE	Bad-smelling meat [1]
*Bradypus variegatus* (Schinz, 1825)		LC	TS	Bad-smelling meat [1]
*Cuniculus paca* (Linnaeus, 1766)		LC	TE and TS	Meat considered fatty and not consumed by Adventist Christians [1, 7]
*Dasypus novemcinctus* (Linnaeus, 1758)		LC	TE	Bad-smelling meat [1]
*Eira barbara* (Linnaeus, 1758)		LC	TE	It is believed that the taste of the meat is sweet because the animal feeds on honey [1]
*Mazama gouazoubira* (G. Fischer [von waldheim], 1814)		LC	TE	Meat causes swelling of the eyes and stomach and causes dizziness [1]
*Nasua nasua* (Linnaeus, 1766)		LC	TE	It has a bad smell [1]
*Pecari tajacu* (Linnaeus, 1758)		LC	TS and TE	Considered unpleasant, it aggravates inflammation [1, 7]
*Saimiri sciureus* (Linnaeus, 1758)		LC	TE	Bad-smelling meat [1]
*Sapajus apella* (Linnaeus, 1758)		LC	TE	Bad-smelling meat [1]
*Alouatta seniculus* (Linnaeus, 1766)		NT	TE	Bad-smelling meat [1]
*Leopardus wiedii* (Schinz, 1821)		NT	TE	Meat with a bad smell and due to the generalist diet of the animal [1]
*Panthera onca* (Linnaeus, 1758)		NT	TE	Consumption can cause headache, swelling and dizziness [1]
*Tapirus terrestres* (Linnaeus, 1758)		VU	TE	Fatty meat and aggravates inflammation [1, 7]
*Tayassu pecari* (Link, 1795)		VU	TE	Fatty meat and aggravates inflammation [1, 7]
*Trichechus inunguis* (Natterer, 1883)		VU	TE	Avoided due to hybrid character (aquatic mammal) [1]
*Dasyprocta leporina* (Linnaeus, 1758)		LC	TS	Not consumed by Adventist Christians [7]
*Trichechus manatus* (Linnaeus, 1758)	Venezuela/South America	VU	TE	Indigenous tribes in the Amazon believe that they are people in the form of the animal [34]
*Manis (Smutsia) temminckii* (Smuts, 1832)	South Africa/Africa	VU	TE	Cultural beliefs can increase the demand for the animal or protect it [42]
*Pteropus tonganus* (Quoy and Gaimard, 1830)	Niue/Oceania	LC	TH	The animal occurs in protected areas [43]
*Birds*				
*Gallus Gallus* (Linnaeus, 1758)	Bangladesh/Asia	NE	TE	Sacred animal [39]
*Aquilla clanga* (Pallas, 1811)		VU	TE	Sacred animal [39]
*Psophia crepitans* (Linnaeus, 1758)	Brazil/South America	LC	TE	Avoided as food for cultural reasons [1]
*Mitu tuberosum* (Spix, 1825)		NT	TS	Not consumed by Adventist Christians [7]
*Psophia viridis* (Spix, 1825)		VU	TS	Not consumed by Adventist Christians [7]
*Reptiles*				
*Eretmochelys imbricata* (Linnaeus, 1766)	Gana/Africa	CR	TE	Cultural beliefs protect the species [44]
	Brazil/South America		TE	Very “strong” meat is avoided by communities [31]
*Chelonia mydas* (Linnaeus, 1758)	Brazil/South America	EN	TE	Very “strong” meat is avoided by communities [31]
*Malayopython reticulatus* (Schneider, 1801)	Indonesia/Asia	LC	TE	Cultural beliefs protect the animal [27]
*Varanus salvator* (Laurenti, 1768)		LC	TE	Cultural beliefs protect the animal [27]
*Varanus bengalensis* (Daudin, 1802)	Bangladesh/Asia	NT	TE	Sacred animal [39]
*Chelonoidis carbonarius* (Spix, 1824)	Brazil/South America	EN	TE and TS	Avoided due to the eating habits of the species, Adventist Christians do not consume; can cause discomfort to those who eat [1, 7, 14]
*Chelonoidis denticulatus* (Linnaeus, 1766)	Brazil/South America	EN	TE and TS	Avoided due to the eating habits of the species, Adventist Christians do not consume; can cause discomfort to those who eat [1, 7, 14]
	Peru/South America		TE	Cultural beliefs protect the species [32]
*Chelus fimbriatus* (Schneider, 1783)	Brazil/South America	LC	TE	They are avoided due to the feeding habits of the species and because they are similar to snakes [1]
*Mesoclemmys gibba* (Schweigger, 1812)	Peru/South America	NE	TE	Avoided due to smell [32]
*Mesoclemmys raniceps* (Gray, 1856)	Brazil/South America	EM	TS	May cause discomfort to those who eat [14]
*Rhinemys rufipes* (Spix, 1824)		NT		May cause discomfort to those who eat [14]
*Caretta caretta* (Linnaeus, 1758)	Gana/Africa	VU	TE	Cultural beliefs protect the species [44]
	Brazil/South America		TS	Meat is considered “strong,” people in a state of vulnerability should avoid [31]
*Dermochelys coriacea* (Vandelli, 1761)	Brazil/South America	VU	TS	Meat is considered “strong,” people in a state of vulnerability should avoid [31]
*Lepidochelys olivacea* (Eschscholtz, 1829)	Gana/Africa	VU	TE	Cultural beliefs protect the species [44]
*Peltocephalus dumerilianus* (Schweigger, 1812)	Brazil/South America	VU	TS	Meat can cause allergies, inflammations, stains and irritations [14]
*Podocnemis unifilis* (Troschel, 1848)	Peru/South America	VU	TE and TS	Only adults can consume parts of it [32]
*Fish*				
*Arapaima gigas* (Schinz, 1822)	Brazil/South America	DD	TE	High concentration of proteins and fats can be harmful [1]
*Acanthocybium solandri* (Cuvier, 1832)		LC	TS	High concentration of proteins and fats can be harmful [38]
*Bagre bagre* (Linnaeus, 1766)		LC	TS	They are prohibited for people in states of vulnerability [25]
*Caranx hippos* (Linnaeus, 1766)		LC	TE	High concentration of proteins and fats can be harmful [4]
*Electrophorus electricus* (Linnaeus, 1766)		LC	TE	Rejected for unpleasant appearance [1]
*Euthynnus alletteratus* (Rafinesque, 1810)		LC	TE	High concentration of proteins and fats can be harmful [4, 30]
*Hoplerythrinus unitaeniatus* (Spix and Agassiz, 1829)		LC	TE	Meat is considered sweet [1]
*Hoplias brasiliensis* (Spix and Agassiz, 1829)		LC	TS	Pregnant women should avoid consumption [45]
*Micropogonias furnieri* (Desmarest, 1823)		LC	TS	Meat is considered “strong,” and vulnerable people should avoid consumption [30]
*Mugil curema* (Valenciennes, 1836)		LC	TE	Greasy [4]
*Oxydoras niger* (Valenciennes, 1821)		LC	TS	It can bring discomfort to those who consume it [2]
*Prochilodus brevis* (Steindachner, 1875)		LC	TS	Pregnant women should avoid consumption [45]
*Satanoperca lilith* (Kullander and Ferreira, 1988)		LC	TE	Meat is considered soft and tasteless [1]
*Scomberomorus brasiliensis* (Mitchhill, 1815)		LC	TS and TE	High concentration of proteins and fats can be harmful [30]
*Scomberomorus cavala* (Cuvier, 1829)		LC	TS and TE	High concentration of proteins and fats can be harmful [30]
*Semaprochilodus brama* (Valenciennes, 1850)		LC	TS and TE	High concentration of proteins and fats can be harmful [30]
*Sternarchorhynchus axelrodi* (de Santana and Vari, 2010)		LC	TE	Rejected by appearance [1]
*Thunnus albacares* (Bonnaterre, 1788)		LC	TS and TE	High concentration of proteins and fats can be harmful [38]
*Trichiurus lepturus* (Linnaeus, 1758)		LC	TS and TE	High concentration of proteins and fats can be harmful [4, 30]
*Zungaro zungaro* (Humboldt, 1821)		LC	TS and TE	High concentration of proteins and fats can be harmful [11]
*Asterophysus batrachus* (Kner, 1858)		EN	TE	Rejected by appearance [1]
*Astronotus crassipinnis* (Heckel, 1840)		EN	TE	Meat is considered soft and tasteless [1]
*Astronotus ocellatus* (Agassiz, 1831)		EN	TS	Prohibited for women during the puerperium, it can cause spots on the woman or the baby [1]
*Brachyplatystoma filamentosum* (Lichtenstein, 1819)		EN	TS and TE	High concentration of proteins and fats can be harmful [2, 4]
*Brachyplatystoma rousseauxii* (Castelnau, 1855)		EN	TS	High concentration of proteins and fats can be harmful [2]
*Calophysus macropterus* (Lichtenstein, 1819)		EN	TS	High concentration of proteins and fats can be harmful [35]
*Cichla ocellaris* (Bloch and Schneider, 1801)		EN	TS	Pregnant women should avoid consumption [45]
*Cichla temensis* (Humboldt, 1821)		EN	TE and TS	Meat spoils quickly [1]
*Crenicichla lenticulata* (Heckel, 1840)		EN	TS	High concentration of proteins and fats can be harmful [1]
*Hoplias malabaricus* (Bloch, 1794)		EN	TS and TE	Pregnant women should avoid consumption; greasy [1, 35, 45]
*Hoplosternum littorale* (Hancock, 1828)		EN	TS	High concentration of proteins and fats can be harmful [2]
*Leporinus fasciatus* (Bloch, 1794)		EN	TS	Prohibited for women during the puerperium, it can cause spots on the woman or the baby [1]
*Mugil gaimardianus* (Desmarest, 1831)		EN	TS	Certain people cannot consume [25]
*Myleus rubripinnis* (Müller and Troschel, 1844)		EN	TS	Prohibited for women during the puerperium, it can cause spots on the woman or the baby [1]
*Phractocephalus hemioliopterus* (Bloch and Schneider, 1801)		EN	TS	High concentration of proteins and fats can be harmful [4, 11]
*Pimelodina flavipinnis* (Steindaschner, 1876)		EN	TS	High concentration of proteins and fats can be harmful [2, 4]
*Pinirampus pirinampu* (Spix and Agassiz, 1829)		EN	TS	High concentration of proteins and fats can be harmful [4, 30, 35]
*Prochilodus nigricans* (Spix and Agassiz, 1829)		EN	TS	High concentration of proteins and fats can be harmful [4, 35]
*Pseudoplatystoma fasciatum* (Linnaeus, 1766)		EN	TS	High concentration of proteins and fats can be harmful [1, 2, 4, 35]
*Pseudoplatystoma punctifer* (Castelnau, 1855)		EN	TS	High concentration of proteins and fats can be harmful [2]
*Pseudoplatystoma tigrinum* (Valenciennes, 1840)		EN	TS	High concentration of proteins and fats can be harmful [2]
*Sternopygus macrurus* (Bloch and Schneider, 1801)		EN	TE	Rejected by appearance [1]
*Trachelyopterus galeatus* (Linnaeus, 1766)		EN	TS	High concentration of proteins and fats can be harmful [2]
*Sardina pilchardus* (Walbaum, 1792)	Portugal/Europe	LC	TS	High concentration of proteins and fats can be harmful [41]

*LST* life history taboos, *TE* specific taboo, *TS* segmental taboo, *TH* habitat taboo, *LC* status least concern, *NT* near threatened, *VU* vulnerable, *EM* endangered and *CR* critically endangered

**Fig. 3:**
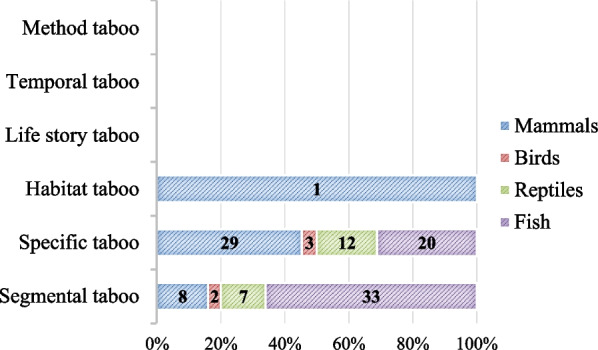
Number of food taboos by animal category

Considering the types of taboos, specific taboos (*n* = 74) and segmental taboos (*n* = 50) showed the highest frequency of animals; the habitat taboo had only one related mammal, and no animals related to the other types of food taboos were recorded (Fig. 3). All the specific and segmental taboos found did not cause the death of the animals. It was also found that the taxonomic category of fish had the highest frequency of segmental taboos, while the class of mammals had a predominance of specific taboos.

It was found that several motivations are pointed out for a species to be considered a food taboo; in this context, the registered species are avoided as food due to the characteristics of the meat (considered sweet, bad taste, unpleasant smell, high protein and fat), cultural beliefs (animals are totemic symbols, sacred, bring bad luck, they are gods), because they aggravate inflammation and cause irritation and for religious reasons.

**Fig. 4:**
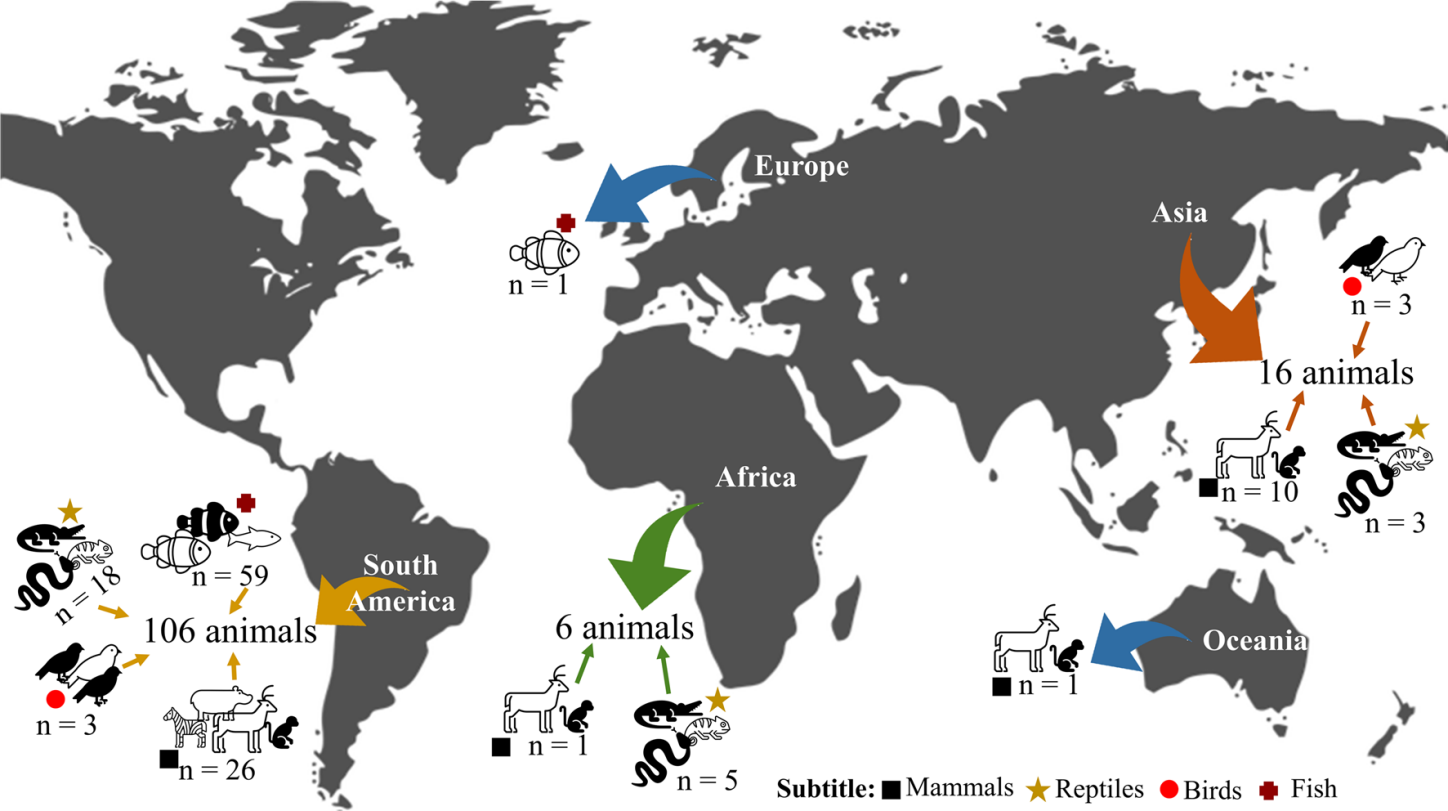
Distribution of taboo species by continent

Analyzing by continent, South America was the continent with the highest number of animals (*n* = 106) (birds: *n* = 3; mammals: *n* = 26; fish: *n* = 59 and reptiles: *n* = 18) mentioned with some type of taboo. None of the described taboos caused the death of animals in this continent. In Africa, only six animals were found, being distributed in the taxa of reptiles (*n* = 5), mammals (*n* = 1). Regarding the types of taboos in the African continent, only specific taboos were found for all animals. Asia recorded 16 taboo animals (birds = 3; mammals = 10 and reptiles = 3). In Europe and Oceania, only two species of animals were described in the studies, one species of fish (with segmental taboo) in the European continent and a mammal in Oceania, respectively (Fig. 4).

The registered animals showed a low rate of endemism, with a total of eight species considered endemic, distributed among six fish (*Pinirampus pirinampu*, *Hoplias malabaricus* and *Cichla ocellaris*, which are threatened with extinction, and *Zungaro zungaro*, *Semaprochilodus brama* and *Hoplias brasiliensis* which are least concern conservation status) and a bird (*Psophia viridis*, vulnerable conservation status), recurrent in Brazil. Only one mammal is considered endemic (*Nycticebus javanicus*, endangered), recurrent in Indonesia. The other species are of continental or cosmopolitan distribution.

As for the type of taboo, the South American continent presented the following types: specific taboo (*n* = 17), segmental taboo (*n* = 88) and habitat taboo (*n* = 1). The class of fish and mammals has the highest number of animals listed by type of taboo, being predominant in the segmental taboo with 54 and 25 animals, respectively. In Asia, specific taboos predominated over the other types of taboos found with eight species in total, followed by segmental taboo (*n* = 4) and habitat taboo (*n* = 4). With respect to the conservation status of the species listed here, it was identified that one species (*Eretmochelys imbricata*) is critically endangered (CR) in terms of conservation status, and 21 are in a state of vulnerability (VU).

## Discussion

Our data indicate that 100 species of vertebrates are related to some type of taboo. Although the patterns of the taboo/species relationship are not clear, it is possible to identify that some animals are rejected as food due to characteristics of the meat, and it is pointed out that consuming some species can aggravate inflammatory processes. At this point, it is necessary to consider that taboos consist of unwritten or defined social rules, generally symbolizing something forbidden and untouchable, without necessarily having a rational explanation [20].

Observing the ecological aspect, the taboos behave like restrictions or rejections that govern attitudes and actions regarding a natural resource, constructed based on the human perception of a certain species. Consequently, species can be avoided because of their behavioral patterns, morphological characteristics, toxicity or simply because they are involved in myths and represent religious symbols, which are part of the cosmology of a population [8, 21]. Examples of species such as *Nycticebus javanicus*, *Funambulus pennantii*, *Pardofelis marmorata* and *Catopuma temminckii* are related in Asia to ancestral relationships, totemic symbols and religious beliefs that protect these species against hunting [28, 29, 46].

It is important to understand how humans seek, obtain and choose food, as food choices can be influenced by individual preferences, ecological, economic, social and cultural factors, as well as dislikes [22]. In this situation, food taboos often limit the use of natural resources and, therefore, have important implications for biodiversity conservation [19, 23, 24].

It is noticeable that taboos are heterogeneously distributed among animal classes, this perspective is possibly related to selective pressures, which led human beings to interact differently with fish, birds and reptiles. About fish, the literature points out many species with an inflammatory potential for humans. It is possible that human populations have developed fish-related taboos to reduce the risks associated with potentially inflammatory foods [4, 25]. Another point is that the rejection for consumption of certain species of fish happens due to the animal's eating conditions, as well as its morphology. For example, species such as poraquê (*Electrophorus electricus*) and the sarapo (*Sternarchorhynchus mormyrus*) are avoided by Brazilian communities because they are like snakes, so in the local perception, they may contain some toxicity [1].

About mammals, the ancestry between humans and other animals of this taxon may be a factor that influences behaviors that originate taboos. As humans recognize characters in common with other mammals, this may lead to dietary restrictions for animals with anthropomorphic characteristics. Traditional peoples of China tend to avoid the Gibbon (*Hoolock tianxing*) as food, due to the belief that the species is “ancestors of people” [26]; it is also found that indigenous peoples of India do not hunt or consume any primates, due to the belief that primates were their ancestors and, therefore, are religious symbols [27]. In this way, shared ancestry, religious symbols and the belief that the species causes or intensifies inflammation can make a species taboo [4, 14, 25–32].

The taboos associated with reptiles and birds report situations of restriction to the meat of these animals due to sacred contexts or potential inflammation. Regarding reptiles, the emergence of taboos associated with these animals may be related to the feeling of fear. Most likely, humans' fear of reptiles is related to genes that arose in ancient lineages of mammals that were preyed on by snakes. Thus, the human feeling of fear is associated with these genes, possibly favoring the survival capacity of *Homo sapiens* against animals with some risk potential, such as snakes [50–52].

About the taboos related to birds, the human feeling about birds is directly associated with the beauty of these animals. Birds are seen by humans as beautiful animals due to their coloration [53]. Colors such as blue and yellow are seen, especially in birds, as elements that enhance beauty [54]. Possibly, this feeling influences a low number of birds used for food and, consequently, fewer food taboos. Additionally, the taboos assigned to birds that have been listed here are related to restrictions constructed by local sacred aspects. It is also necessary to consider that this taxon is directly linked to smuggling, in which several birds are sold in Brazil and in the world, causing birds to be incorporated into pet and trade categories [55, 56].

Taboos can be classified in a utilitarian way, such as temporary (segmental) taboos that are restricted to certain periods of life, regulating the use of a resource according to age, gender, social condition and other specific conditions; and permanent (specific) that extend throughout life [19]. As for the variation in the types of taboos, the segmental taboos predominated in relation to the other types of taboos observed in the studies. Many of these segmental taboos are associated with the inflammatory potential of meat. These animals are known as “reimosos” in South America. The word “reima” comes from the Greek “rheum” which means “viscous fluid” and aims to classify the degree of safety of wild and domestic animals for consumption [1].

Creamy or “heavy” foods, for traditional populations, tend to provoke or aggravate inflammatory processes, tending to be avoided by people in physical states of liminality, initiated in some ritual, people with illnesses, menstrual period and postpartum [12, 33]. In our study, we found 50 cases of taboos referring to “heavy animals,” many of which were described as “heavy meat” animals capable of causing infections, being foods to be avoided mainly by women during pregnancy, puerperium or menstruation. This perspective is recurrent in riverside communities in the Amazon (Basil), where some reptiles such as the Jabutis (*Peltocephalus dumerilianus*), (*Mesoclemmys raniceps*) and the jabuti-tinga (*Chelonoidis denticulatus*) are not eaten because they are oily, because they are “offensive to anyone eats,” causing “allergic reactions” [14]. Several other cases of segmental taboos are cited in this review [1, 4, 11, 14, 31, 34–38]. These examples of segmental taboos point out how cultural factors and the phases of a person's natural life cycle can interfere in the dynamics of animal consumption in a community, and this instrument ends up being an important factor for the conservation of animal species.

Specific taboos are mostly related to religious factors and folk beliefs. In a case study, it is seen that the capture and consumption of primacy *Nycticebus javanicus* is prohibited because, according to villagers, taking and keeping this species in homes can bring unhappiness and bad luck [28]. On the other hand, in India, felines such as Capped Langur (*Trachypithecus pileatus*)*,* Asian golden cat* (Catopuma temminckii*)*, cat-*marbled* (Pardofelis marmorata*) and the tiger (*Panthera tigris*) are seen as animals that bring luck, because they are related to sacred institutions and cannot be hunted [27].

Habitat taboos are also considered a type of permanent taboo. This type of taboo was characterized by restrictions on hunting in places considered sacred. These places, because they are surrounded by symbology and spirituality, serve as a sanctuary for animals, thus being an important conservation factor. According to local beliefs, people who hunt in sacred places can suffer both divine and popular punishments [39]. Janaki et al. [27] point out that habitat taboos can help in the conservation of wild animals by providing refuges. Habitat taboos are recurrent in continents such as South America, Asia and Oceania, and these sacred reserves help government institutions to institutionalize places as biodiversity conservation areas, making them heritage protected by law.

The studies found are mostly from South America, reinforcing the perspective that this continent is one of the main scientific productions related to Ethnobiology [40]. It can be noticed that regarding taboo game species in South America, the vast majority of studies are focused on the fish group, with case studies being carried out with indigenous and riverside peoples, mainly in the Brazilian Amazon, in addition to caiçaras (mixture ethnocultural heritage of indigenous, European and African peoples) from the coastal portion of Brazil [1, 2, 4, 11, 25, 30, 38]. On the other hand, no studies were found that portrayed taboos associated with fish in Asia, Oceania and Africa. And only one study was found in Europe [41].

The greatest restriction for fish consumption in South America was due to the potential to cause inflammation, the feeding habits of these animals, in addition to the morphological similarities with snakes for some species [4, 25, 32]. In Asia, Africa, Oceania and Europe, it is noticeable that the taboos are similar, since most food restrictions are based on spirituality, where species, mainly mammals and reptiles, are prohibited so that the hunter/consumer does not suffer “punishments,” divine powers or punishments in their village/tribe [27, 28, 39, 42, 43].

By observing the behavior of taboos within the socioecological systems present in this review, it was found that food taboos have a positive effect on fauna conservation. This is because, even if unintentionally, the people involved end up acting in favor of the conservation of the species, either by restricting the consumption of “loaded” meat that can cause illness or by situations associated with the sacred place that can result in punishments for those who consume [14, 27, 43].

The literature directly discusses the effect of taboo on fauna conservation [13, 21]. The compilation of data on taboos across the planet corroborates this perspective, as the data collected here show that food taboos have a positive effect on animal conservation, as of the 100 species listed under the effects of food taboos, 99 have taboos with positive effects for these species. These results show how taboos play a fundamental role in conservation and are often neglected by representations of formal institutions.

Analyzing the conservation status of the species listed here, we observe that the species classified as critically endangered (CR) in the IUCN list, as is the case of the hawksbill turtle (*Eretmochelys imbricata*) and the small primate the slow loris (*Nycticebus javanicus*) have taboos that reduce access by humans. We can presume that these species, without local taboos, could be susceptible to a decrease in population density in several regions of their occurrence [28, 31, 44].

However, it is important to consider the limitations of the effect of food taboos within a conservationist perspective [21, 57]. Some species may present local taboos and have their consumption avoided, but form part of the diet of other human populations. For example, the present study shows that Tayassu pecari, Pecari tajacu and Nasua nasua have a record of food taboos in Brazil; however, it is used in food in different parts of northeastern Brazil [58, 59]. Additionally, species such as Mazama americana, Mazama gouazoubira, Dasypus novemcinctus and Cuniculus paca have food taboos in Argentina but are preferred items in food in some locations in Brazil [55, 59].

Considering that habitat loss (because of urbanization and agribusiness) [60, 61] directly impacts wildlife, the existence of food taboos, even at the local level, plays an important role in conservation. If we consider that the food taboo has a local effect, the absence of these social rules could trigger greater pressure on certain species of animals, as their consumption would be widely spread. In this way, a species of animal avoided by a certain social group tends to have a higher population density at the local level, thus contributing to conservation. For example, in a study on sacred groves, it is demonstrated that the taboo of habitat serves to regulate the use of natural resources, being recognized by traditional communities as more efficient than areas of environmental protection [57]. Segmental taboos have also been identified as important wildlife managers, since they reduce the number of people who consume the resource [30].

The data collected here show that there are still few studies on food taboos and their consequences for preserving fauna. Thus, any strong conclusion about the role of taboos in conservation is still premature. However, it is possible to use these data and incorporate them into strategies to support fauna conservation. Taboos associated with the sacred are efficient mechanisms in the conservation of fauna. In a case study in Ghana (Africa), it is pointed out that among a community of turtles such as *E. imbricata*, *Dermochelys coriacea, Lepidochelys olivaceae* and *Chelonia mydas* are not hunted, due to local belief that these turtles were sighted saving ancestors of the population during a war against the Ashanti empire (an important ethnic group in Ghana). Therefore, residents of this village are prevented from consuming meat from these reptiles [44]. In the Brazilian Amazon, the taboo exerts a positive force (conservation) on species such as *Tapirus terrestres*, *Tayassu fishermen*, *Fishermen steal* and *Ateles chamek* which are avoided by indigenous peoples of the lower Madeira River, as they are considered to aggravate inflammation [7].

The consensus among studies is that animals considered taboo tend to be preserved, and this can positively impact the population dynamics of these species. It is estimated that the existence of taboos can reduce the pressure exerted on some species by up to 80%, since taboos reduce the number of people sharing the resource [4, 13, 14]. At this point, it was identified that only one work points to a negative relationship of taboos associated with wild species; it was found that in Oceania, flying fox hunting (*Tongan priest*) is intensified, due to the belief that the population of this species is infinite within a sacred area, so hunting the species in other areas does not impact the population of the animal [43].

Considering the types of taboos, it is observed that the specific and habitat taboos, as they are permanent, contribute to the formulation of laws and other regulations to prevent the hunting of different species of animals [57], showing the importance of the taboo even for formal institutions as technical and legal mechanisms for the conservation of species, corroborating the study by Nijman and Nekaris [28], which points out that species-specific taboos may have important ecological ramifications for the protection of threatened populations.

## Final considerations

The present study covered the status of current knowledge about food taboos associated with wildlife in the world. It is noticeable that taboos have a considerable effect on animal conservation, as the social restrictions imposed by taboos effectively contribute to the local conservation of species. Even considering the importance of taboos for socio-biodiversity, there are still crucial gaps on this topic, showing that the topic “food taboo” is often neglected or little explored within socio-ecological systems.

From this study, it is evident the need to develop research to elucidate the mechanisms that favored the emergence of taboos. Undoubtedly, investigating human evolutionary history and foraging in the environment is an interesting way to identify what favored the emergence of taboos. Additionally, food taboos are important for maintaining the population of species on different continents. It is also important to emphasize that due to the inclusion and exclusion criteria of this research, data on other species and types of food taboos have been subtracted, so the number of species under the effects of food taboos may be even greater.

In this way, we point out that new studies should be designed to include objectives and metrics to analyze food taboos, seeking to understand how taboos arise and remain qualitatively and quantitatively within human populations. We also indicate that considering food taboos in environmental management plans can contribute significantly to the conservation of certain species.

## Supplementary Information

**Additional file 1**. General information about articles involving “taboos” included in the systematic review

## Data Availability

All data generated or analyzed during this study are included in this published article.
