# Supplementary material for: Food taboos and animal conservation: a systematic review on how cultural expressions influence interaction with wildlife species
Source: J Ethnobiol Ethnomed. 2023 Jul 15;19:31. doi: 10.1186/s13002-023-00600-9 (PMC10349426; doi:10.1186/s13002-023-00600-9)
Supplement: Supplementary file 1 — Additional file 1. General information about articles involving “taboos” included in the systematic review [file 13002_2023_600_MOESM1_ESM.docx]

| Additional file 1: General information about articles involving “taboos” included in the systematic review | | | | | | |
| --- | --- | --- | --- | --- | --- | --- |
|  | **Author** | **Title** | **Research object** | **Country** | **Continent** | **Number of species under taboo** |
| 1. | Alexander et al. [44] | The role of taboos in the protection and recovery of sea turtles. | Recognize the presence of taboos about sea turtles in fishing communities and verify how traditional practices influence their conservation. | Enough | Africa | 5 |
| 2. | Baiyewu et al. [42] | Ethnozoological survey of traditional uses of Temminck’s ground pangolin (*Smutsia temminckii*) in South África. | To determine the impact and demand for the terrestrial pangolin in rural South Africa and to examine the uses of this species for traditional cultural and medicinal purposes across the animal's range. | South Africa | Africa | 1 |
| 3. | Bassan et al. [38] | Recursos pesqueiros utilizados pela comunidade local do Arquipélago de Fernando de Noronha (PE, Brasil): preferências, tabus alimentares e uso medicinal. | To analyze the local knowledge and eating habits of people from Noronha, their preferences, and dislikes regarding the consumption of fish from the Archipelago of Fernando de Noronha/PE. | Brazil | South America | 2 |
| 4. | Batista et al. [45] | Etnotaxonomia e tabus alimentares dos pescadores artesanais nos açudes Araras e Edson Queiroz, bacia do rio Acaraú, Ceará, Brasil. | Evaluate the ethnotaxonomy and food taboos of artisanal fishermen from public dams Araras and Edson Queiroz (Ceará, Brazil). | Brazil | South America | 4 |
| 5. | Begossi e Braga [11] | Food taboos and folk medicine among fishermen from the Tocantins river (Brazil). | Analyze fish utilization and understand why some available fish are harvested while others are rejected as food. | Brazil | South America | 5 |
| 6. | Begossi et al. [35] | Uses of fish and game by inhabitants of na extractive reserve (upper Juruá, Acre, Brazil). | Show the different interpretations of food taboos observed in tropical societies, including their association with the availability of resources. | Brazil | South America | 5 |
| 7. | Begossi et al. [4] | Food chain and the reasons for fish food taboos among Amazonian and atlantic forest fishers (Brazil). | To reveal that in the Amazon and the Atlantic Forest coast, fish food taboos, or food prohibitions during illness, are associated with carnivorous fish, especially piscivores. | Brazil | South America | 10 |
| 8. | Braga and Schiavetti [31] | Attitudes and local ecological knowledge of experts fishermen in relation to conservation and Bycatch of sea turtles (reptilia: testudines), Southern Bahia, Brazil. | Investigate ecological knowledge of fishermen about sea turtles and attitudes towards conservation and their bycatch. | Brazil | South America | 4 |
| 9. | Braga et al. [41] | Sharing fisher’s ethnoecological knowledge of the european pilchard (*Sardina pilchardus*) in the westernmost fishing community in Europe. | Register the ethnoecological knowledge of *Sardina pilchardus* in the traditional fishing community of Peniche, Portugal | Portugal | Europe | 1 |
| 10. | Braga et al. [2] | Preferências e tabus alimentares no consumo de pescado em Santarém, Brasil. | Record the current use of fishing resources by residents of the city of Santarém, in the lower Amazon, and analyze aspects associated with avoiding or increasing the consumption of certain species. | Brazil | South America | 8 |
| 11. | Brooke and Tschapka [43] | Threats from overhunting to the flying fox, *Pteropus tonganus*, (Chiroptera: Pteropodidae) on Niue Island, south pacific Ocean. | Estimate the population size of *Pteropus tonganus* in Niue, to assess the number of flying foxes shot during hunting and to determine whether the current level of hunting is sustainable. | Niue | Oceania | 1 |
| 12. | Camino et al. [37] | Relations with wildlife of wichi and criollo people of the dry Chaco, a conservation perspective. | Assist in the development of actions for the conservation of wild species in this region, focusing on the perceptions and relationships of Wichís and Creoles with wildlife. | Argentina | South America | 2 |
| 13. | Chowdhury et al. [39] | Dietetic use of wild animals and traditional cultural beliefs in the Mro community of Bangladesh: an insight into biodiversity conservation. | Explore local culture and its impact on local biodiversity in the highland forests of southeastern Bangladesh, based on a study of the Mro tribe, the country's oldest aboriginal group. | Bangladesh | Asia | 4 |
| 14. | Ferronato and Cruzado [32] | Uses, beliefs, and conservation of turtles by ashaninka indigenous people, Central Peru. | Document the occurrence of species, taboos and uses of turtles by Ashaninka communities in central Peru and explore how their traditional knowledge can help protect turtles. | Peru | South America | 2 |
| 15. | Hanazaki and Begossi [25] | Catfish and mullets: the food preferences and taboos  of caiçaras (Southern Atlantic forest coast, Brazil). | To analyze food preferences and taboos on animal protein items among three caiçara communities on the southeastern coast of Brazil. | Brazil | South America | 2 |
| 16. | Janaki et al. [27] | The role of traditional belief systems in conserving biological diversity in the eastern Himalaya ecoregion of Índia. | Evaluate whether the informal aspects adopted by these communities can play a role in maintaining the biological diversity of the region and whether the consideration of traditional beliefs and taboos in conservation planning can influence the conservation of the species. | India | Asia | 5 |
| 17. | Knoop et al. [7] | Age, religion, and taboos influence subsistence hunting by indigenous people of the lower madeira river, Brazilian Amazon. | Evaluate how religious taboos associated with Adventism determine the composition of target taxa for food. | Brazil | South America | 10 |
| 18. | Kushwah et al. [29] | Magico-religious and social belief of tribals of district udaipur, Rajasthan. | Investigate the domesticated and wild species used for spiritual and religious purposes among the tribals of six tehsils of Udaipur district. | India | Asia | 3 |
| 19. | Nijman and Nekaris [28] | Traditions, taboos and trade in slow lorises in sundanese communities in southern java, Indonesia. | To assess whether beliefs, opinions and knowledge about Java slow lorises, *Nycticebus javanicus*, in 12 Sudanese communities in West Java, Indonesia are shared among local residents. | Indonesia | Asia | 1 |
| 20. | O’shea et al. [34] | Distribution, status, and traditional significance of the west indian manatee trichechus manatus in Venezuela. | Gather information on the traditional significance of manatees in Venezuela in relation to their conservation needs. | Venezuela | South America | 1 |
| 21. | Pezzuti et al. [14] | Uses and taboos of turtles and tortoises along rio Negro, Amazon basin. | Investigate how people living in the Rio Negro area use Amazonian turtles, including issues of consumption, preferences, restrictions, segmental taboos, harmfulness, medicinal use and sale. | Brazil | South America | 5 |
| 22. | Ramires et al. [30] | The use of fish in Ilhabela (São Paulo/Brazil): preferences, food taboos and medicinal indications. | Analyze the preferences, taboos and medicinal indications of the fish and, in this way, represent the interactions of the fishermen with the fishing resources, aiming to understand the biological and cultural aspects involved. | Brazil | South America | 6 |
| 23. | Silva [1] | Comida de gente: preferências e tabus alimentares entre os ribeirinhos do médio rio negro (Amazonas, Brasil). | To analyze aspects related to food choices and aversions among riverside populations settled on the Rio Negro (Amazonas, Brazil). | Brazil | South America | 39 |
| 24. | Uyeda et al. [46] | The role of traditional beliefs in conservation of herpetofauna in banten, Indonesia. | Collaborate with specific knowledge about local perspectives and discuss the role of traditional beliefs in the conservation of wildlife populations in Indonesia. | Indonesia | Asia | 2 |
| 25. | Zhang et al. [26] | Influence of traditional ecological knowledge on conservation of the  skywalker Hoolock gibbon (*Hoolock tianxing*) outside nature reserves. | Examine whether the traditional ecological knowledge - TEK (knowledge, practice and belief) of local communities across the gibbon distribution (*Hoolock Tianxing*) in Yunnan Province, China, has helped keep gibbon populations out of nature reserves by suppressing local hunting pressure on gibbons. | China | Asia | 1 |
